# Curcumin-Induced Vacuolizing Stress Reveals a Vulnerability Associated with NF1 Loss in Schwann Cells

**DOI:** 10.3390/cells15201904

**Published:** 2026-10-20

**Authors:** Assunta Maria Iazzetta, Francesca Sardina, Cinzia Rinaldo

**Affiliations:** Institute of Molecular Biology and Pathology, National Research Council, 00185 Rome, Italy; am.iazzetta@outlook.it (A.M.I.); francesca.sardina@cnr.it (F.S.)

**Keywords:** NF1, Schwann cells, curcumin, vacuolizing stress, clonogenic recovery

## Abstract

Neurofibromatosis type 1 (NF1) predisposes to peripheral nerve sheath tumors arising from Schwann-lineage cells with biallelic NF1 loss and remains in need of biologically informed strategies compatible with chronic disease management. Curcumin has attracted interest in NF1, but its effects in disease-relevant cells remain unclear. Here, we investigated curcumin responses in patient-derived NF1-deficient Schwann cells. Curcumin induced the progressive accumulation of cytoplasmic vacuoles resembling those elicited by a reference methuosis inducer, without prominent evidence of canonical apoptotic features. Vacuolization was associated with impaired long-term clonogenic recovery, indicating a durable loss of proliferative fitness. NF1-deficient Schwann cells displayed lower levels of NRF2, thioredoxin, and SOD1 than NF1-proficient cells under basal and curcumin-treated conditions, defining a distinct NF1-associated antioxidant-response context. Consistent with a functionally relevant redox-sensitive component, N-acetylcysteine pretreatment reduced curcumin-induced vacuolization to near-basal levels and substantially improved clonogenic recovery after drug washout. NF1-proficient Schwann cells showed an attenuated vacuolizing response, whereas acute NF1 silencing in hTERT RPE-1 cells enhanced curcumin-induced vacuolization in an independent cellular background. Together, these findings identify an NAC-sensitive vacuolizing response associated with loss of cellular fitness as a previously underappreciated vulnerability of NF1-deficient cells and provide a disease-relevant framework for understanding curcumin responsiveness in NF1.

## 1. Introduction

Neurofibromatosis type 1 (NF1) is a common autosomal dominant tumor-predisposition syndrome in which affected individuals carry a germline heterozygous pathogenic variant in the NF1 gene encoding neurofibromin [1,2]. Among its most characteristic manifestations are benign peripheral nerve sheath tumors, including cutaneous and plexiform neurofibromas, which impose a chronic disease burden and remain in need of biologically informed and well-tolerated strategies suitable for chronic disease management. Although patients are constitutionally NF1+/−, neurofibromas arise when a Schwann-lineage cell undergoes somatic inactivation of the remaining NF1 allele, resulting in biallelic NF1 loss within the neoplastic Schwann-cell compartment. Thus, NF1-deficient Schwann cells represent the disease-relevant cellular state underlying neurofibroma formation [3,4].

Beyond its canonical role as a negative regulator of RAS signaling, neurofibromin loss has been associated with broader alterations in cellular stress adaptation, metabolism, and oxidative homeostasis [5,6,7]. Growing evidence further supports a role for neurofibromin in the maintenance of mitochondrial and redox homeostasis, suggesting that NF1-deficient cells may exist in altered stress-buffering states that extend beyond proliferative signaling alone. In line with this view, recent proteomic analyses of NF1-null Schwann cells indicate broad metabolic rewiring, including reduced oxidative phosphorylation, mitochondrial dysfunction, increased glycolytic features, and altered oxidative stress-related programs, consistent with impaired cellular stress buffering [8].

Curcumin is a pleiotropic natural compound with extensive human exposure and reported antioxidant, anti-inflammatory, and anti-tumor activities [9,10,11]. Because of these properties, curcumin has attracted interest in NF1, including in a small clinical nutritional intervention reporting symptom improvement and neurofibroma reduction in affected individuals [12]. However, despite this translational interest, the cellular basis of curcumin responsiveness in NF1-relevant cells remains poorly understood.

Vacuolizing non-apoptotic cell death programs have emerged as biologically relevant stress responses in cancer and disease-associated cellular contexts [13,14]. Among them, methuosis is characterized by the accumulation of large cytoplasmic vacuoles associated with dysregulated endocytic trafficking [15,16,17]. Methuosis was originally described in RAS-activated glioblastoma cells and represents a paradigmatic example of a vacuolizing non-apoptotic response associated with progressive cellular failure [15].

Although methuosis has not been established in Schwann-cell models of NF1, a methuosis-like framework provides a useful context for interpreting prominent vacuolizing non-apoptotic responses in NF1-deficient cells without presupposing a fully defined mechanism.

Here, we sought to characterize the cellular response of NF1-deficient Schwann cells to curcumin exposure and determine whether NF1 loss influences the susceptibility to this response. We found a prominent vacuolizing phenotype with methuosis-like features and examined its relationship with NF1 status, antioxidant-response markers, long-term clonogenic outcomes, and antioxidant modulation.

## 2. Results

### 2.1. Curcumin Elicits a Prominent Vacuolizing Response in NF1-Deficient Schwann Cells

**Figure 1:**
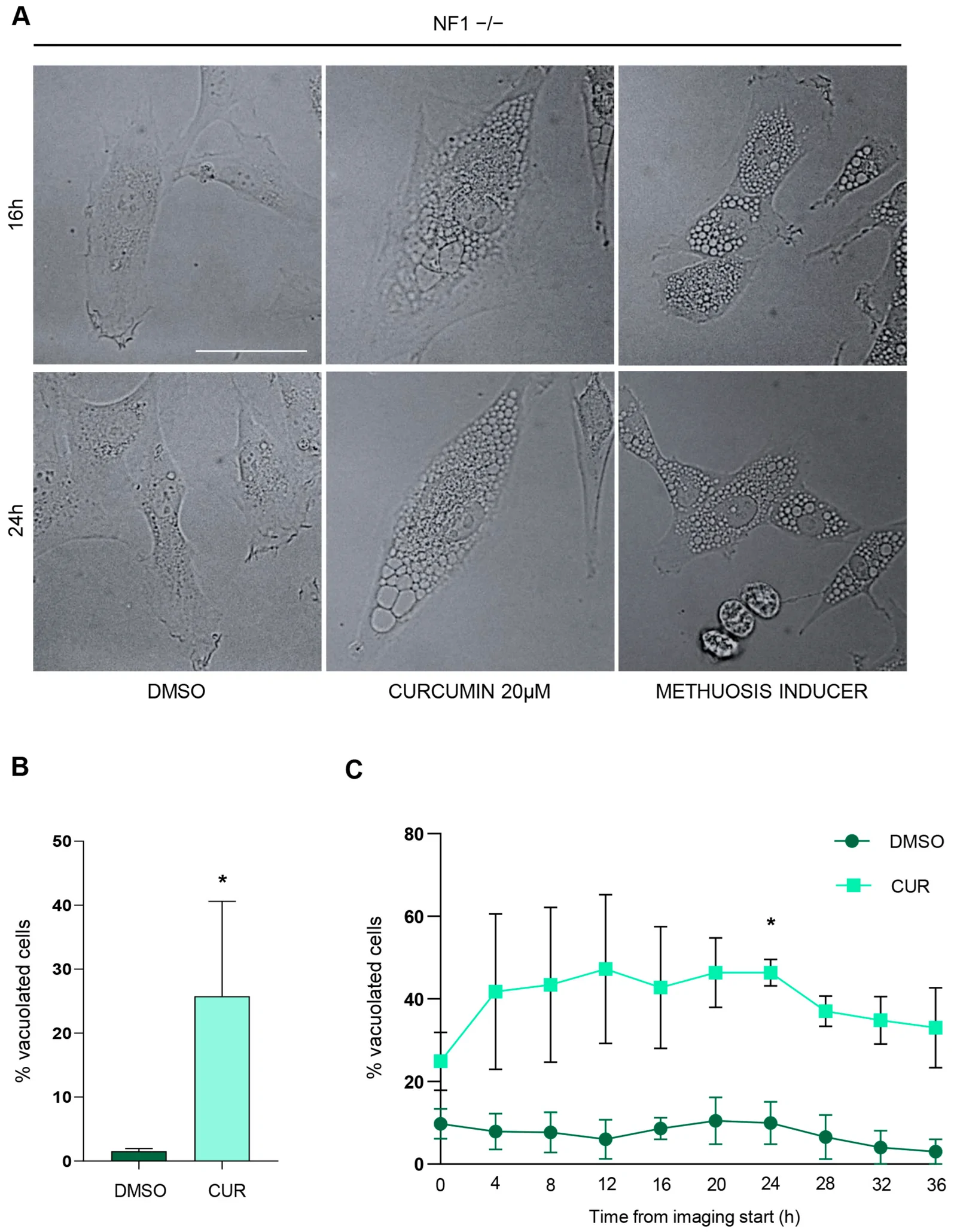
Curcumin elicits a progressive vacuolizing response in NF1-deficient Schwann cells. (**A**) Representative images of patient-derived NF1-deficient Schwann cells treated with vehicle (DMSO), curcumin 20 μM, or the reference methuosis inducer 1 (500 nM) at the indicated time points. Scale bars, 50 μm. The reference inducer was included as a qualitative comparator for vacuolar morphology. (**B**) Quantification of vacuolated NF1-deficient Schwann cells following treatment with vehicle or curcumin 20 μM for 24 h. Data are expressed as the percentage of cells displaying two or more prominent cytoplasmic vacuoles. At least 43 cells per condition were scored in each independent experiment. Data are shown as mean ± SD from three independent experiments. * *p* < 0.047, unpaired *t*-test. (**C**) Time-lapse quantification of vacuolated cells in NF1-deficient Schwann cells exposed to curcumin 20 μM or vehicle. Imaging was initiated 1 h after treatment and the same fields were followed longitudinally within each experiment. The percentage of vacuolated cells was calculated separately for each of three independent biological experiments at every time point. Data are shown as mean ± SEM. At least 63 cells were analyzed per condition across the three experiments. Statistical analysis was performed using multiple *t*-test corrected for multiple comparisons using Holm–Sidak method. * *p* = 0.03.

To investigate the cellular basis of curcumin responsiveness in NF1-relevant cells, we first examined the response of patient-derived NF1-deficient Schwann cells [18] to curcumin exposure. Given the diverse biological activities previously attributed to curcumin [19,20,21,22], we initially evaluated several candidate cellular responses, including apoptotic features and stress-response-associated markers. Under these conditions, the most prominent and reproducible phenotype was the progressive accumulation of large cytoplasmic vacuoles in curcumin-treated NF1-deficient Schwann cells, compared with the low baseline vacuolization observed in vehicle-treated controls (Figure 1A–C; Supplementary Figure S1).

The morphology of curcumin-induced vacuoles closely resembled that observed after treatment with the reference methuosis inducer 1 (HZX-02-059), which was included as a qualitative morphological comparator ([23]; Figure 1A), supporting the interpretation that curcumin elicits a vacuolizing phenotype with methuosis-like features in this cellular context.

Quantitative analysis confirmed a marked increase in the proportion of vacuolated cells and time-lapse imaging further showed accumulation of vacuolated cells following curcumin exposure, while progressive cell detachment affected the number of adherent, scorable cells at later time points (Figure 1B,C). A similar response was observed in an additional NF1-deficient patient-derived Schwann cell line, supporting the reproducibility of this response across independent NF1-deficient Schwann-cell models (Supplementary Figure S1).

### 2.2. Curcumin-Induced Vacuolization Is Associated with Impaired Clonogenic Recovery in NF1-Deficient Schwann Cells

**Figure 2:**
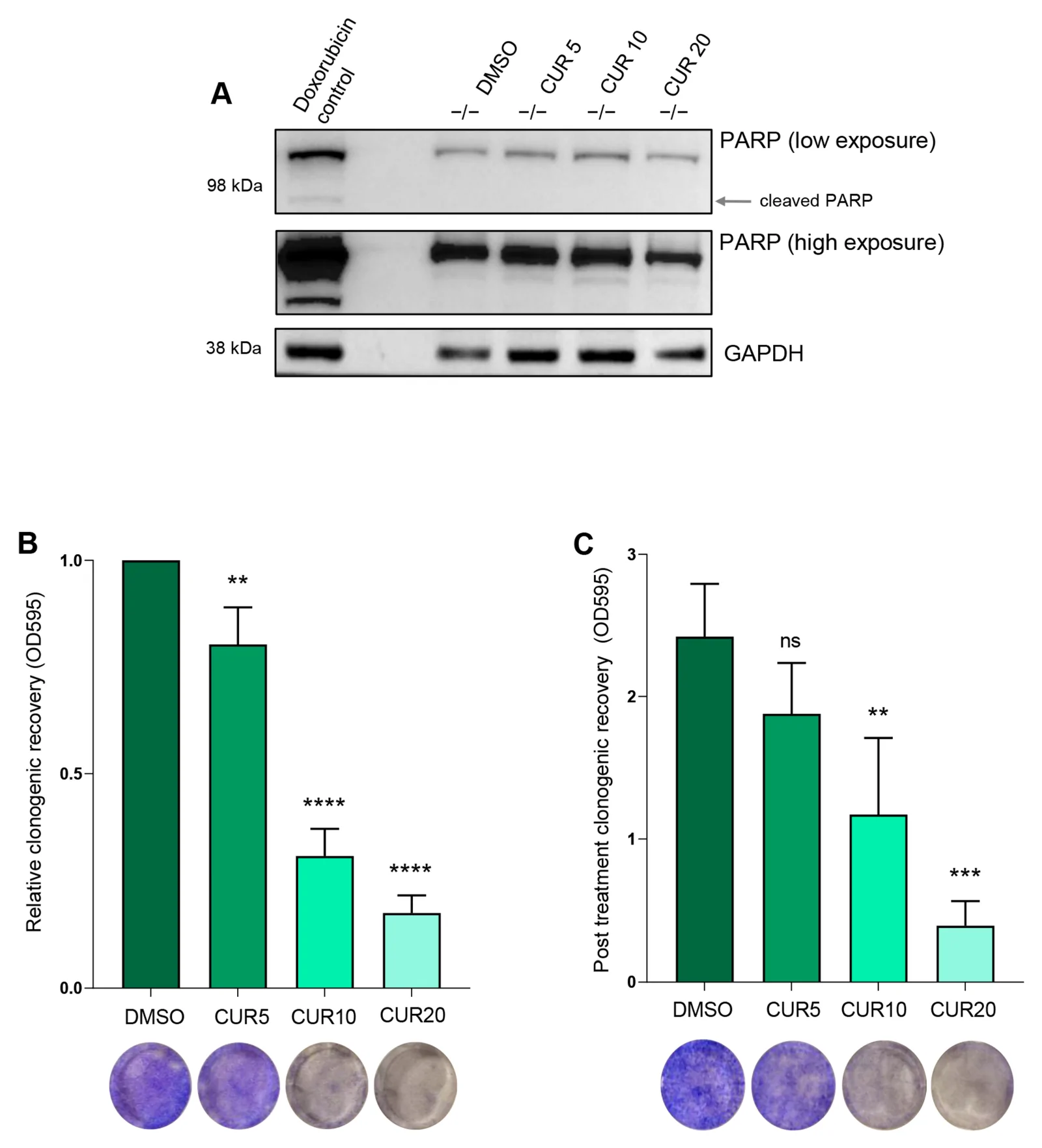
Curcumin exposure impairs long-term recovery of NF1-deficient Schwann cells without prominent PARP processing. (**A**) Representative WB of PARP in NF1-deficient Schwann cells treated with vehicle or curcumin at the indicated concentration for 24 h. A lysate from doxorubicin-treated HCT116 cancer cells was included on the same blot as a positive control for apoptosis to verify PARP cleavage. GAPDH was used as loading control. Data are shown as mean ± SD from three independent experiments. Statistical significance was calculated relative to DMSO-treated controls. ** *p* = 0.007; **** *p* < 0.0001; one-way ANOVA test corrected for multiple comparisons using Dunnett method. (**B**) Quantification and representative images of colony outgrowth following curcumin exposure at the indicated concentrations and replating at a defined cell density. (**C**) Quantification and representative images of post-treatment recovery following curcumin washout and incubation in fresh drug-free medium. Colony outgrowth and post-treatment recovery were assessed by crystal violet staining, and retained crystal violet was quantified by measuring absorbance at 595 nm. Data are shown as mean ± SD from three independent experiments. Statistical significance was calculated relative to DMSO-treated controls. ns, not significant; ** *p* = 0.01; *** *p* = 0.0005; one-way ANOVA test corrected for multiple comparisons using Dunnett method.

We next asked whether the vacuolizing response induced by curcumin in NF1-deficient Schwann cells was associated with measurable functional consequences. Because prominent vacuolization was already evident under conditions that did not produce overt apoptotic morphology, we first assessed early cell viability and apoptosis-associated PARP and caspase-3 processing. At 24 h, curcumin produced only a limited not significant reduction in the MTS-based short-term viability readout, after normalization to DMSO-treated controls (Supplementary Figure S2). Under the same vacuolization-dominant conditions, PARP cleavage nor appreciable accumulation of cleaved caspase-3 was detected in curcumin-treated NF1-deficient Schwann cells (Figure 2A). Together these findings indicate that curcumin-induced vacuolization is not accompanied by clear biochemical evidence of canonical caspase-dependent apoptosis.

Prior exposure to curcumin markedly reduced the clonogenic potential of NF1-deficient Schwann cells (Figure 2B,C). This effect was consistently observed using two complementary long-term assay settings: first, by treating cells, replating defined numbers, and quantifying subsequent colony outgrowth (Figure 2B); and second, by assessing post-treatment recovery after drug washout in fresh curcumin-free medium (Figure 2C). In both settings, curcumin impaired long-term proliferative recovery, indicating that the vacuolizing response is associated with a loss of proliferative fitness.

### 2.3. NF1-Deficient Schwann Cells Show Reduced Levels of Selected Antioxidant-Response Proteins

**Figure 3:**
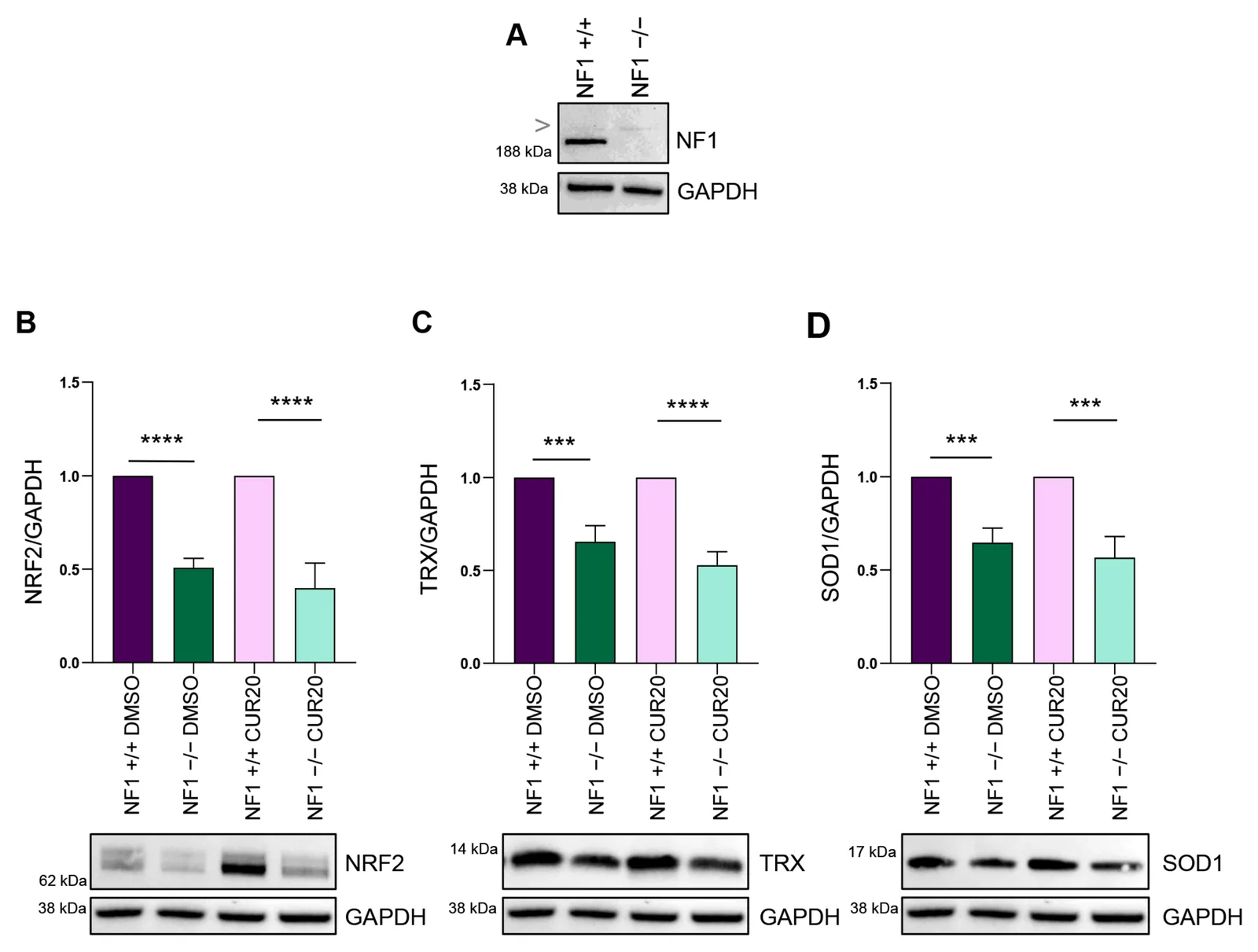
Comparison of selected antioxidant-response proteins in NF1-proficient and NF1-deficient Schwann cells. (**A**) Representative WB of NF1 in NF1-proficient and NF1-deficient Schwann cells. The arrow indicates a non-specific band. (**B**–**D**) Representative WB and corresponding densitometric quantification of NRF2 (**B**), TRX (**C**), and SOD1 (**D**) in NF1-proficient and NF1-deficient Schwann cells treated with vehicle or 20 μM curcumin for 24 h. Protein levels were normalized to GAPDH and expressed relative to NF1-proficient cells. Quantification represents three independent biological experiments. The representative immunoblots shown in panels (**B**–**D**) were selected from the same experiment and therefore share a common GAPDH loading control, which is reported in each panel. Data are shown as mean ± SD from three independent experiments. NRF2 **** *p* < 0.0001; TRX *** *p* = 0.0001, **** *p* < 0.0001; SOD1 *** *p* = 0.0004; *** *p* = 0.0001, one-way ANOVA test, corrected for multiple comparisons using Sidak method.

Given the known ability of curcumin to engage oxidative stress-response pathways, including the KEAP1/NRF2 axis, we next asked whether NF1 status was associated with differences in the levels of selected antioxidant-response proteins in Schwann cells. Under basal conditions, NF1-deficient Schwann cells displayed lower levels of NRF2 and of the antioxidant effectors thioredoxin (TRX) and SOD1 compared with NF1-proficient cells (Figure 3A–D), indicating that the NF1-deficient Schwann-cell background is characterized by reduced expression of selected antioxidant-response markers.

Differences according to NF1 status were also maintained following curcumin exposure. At 24 h after treatment, NRF2, TRX, and SOD1 protein levels remained lower in NF1-deficient than in NF1-proficient Schwann cells (Figure 3B–D).

Overall, these findings indicate that curcumin-induced vacuolization occurs in an NF1-deficient cellular background characterized by lower levels of NRF2, TRX, and SOD1 relative to NF1-proficient Schwann cells, both before and after treatment.

### 2.4. N-Acetylcysteine Markedly Reduces Curcumin-Induced Vacuolization and Improves Post-Treatment Recovery

**Figure 4:**
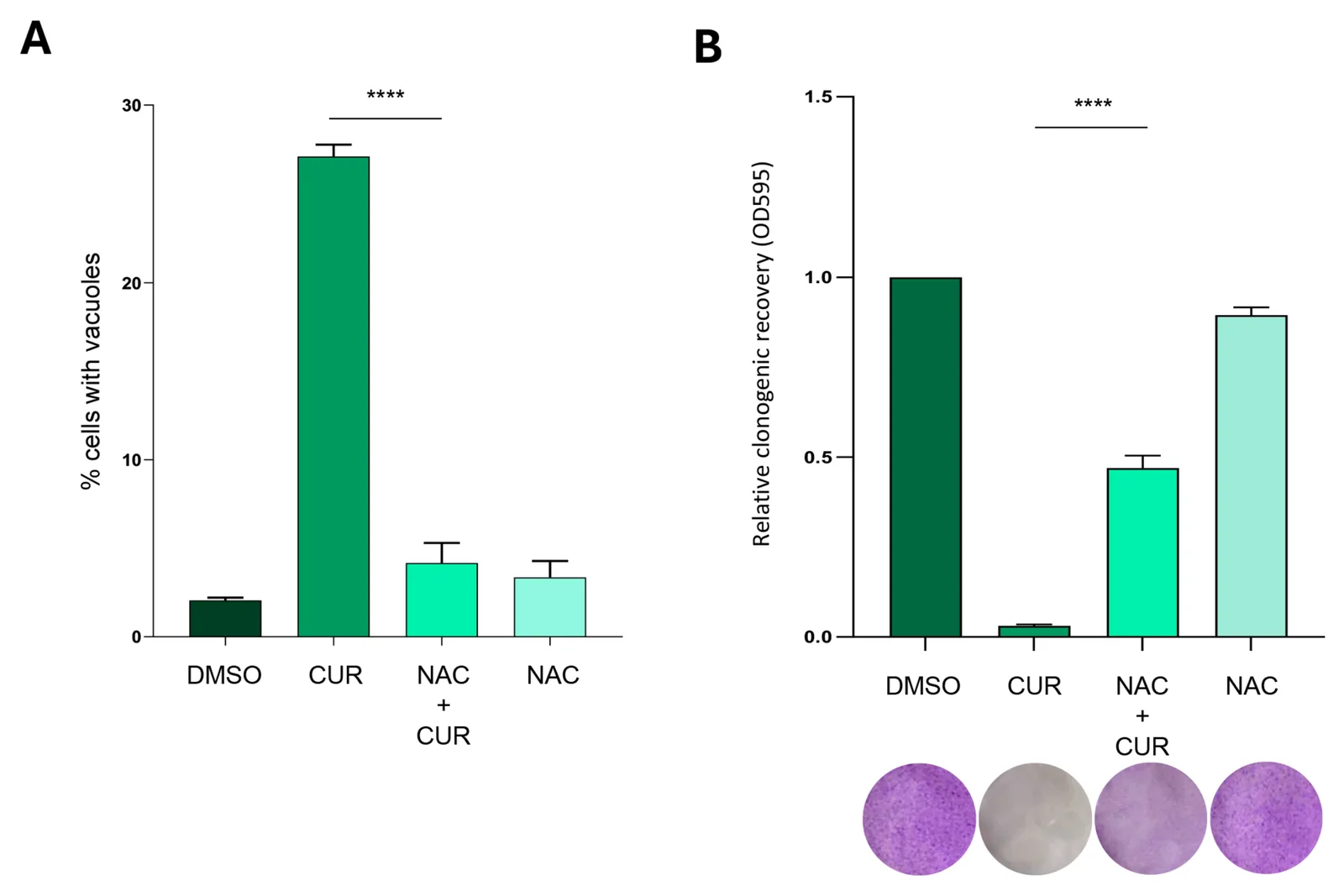
Effects of N-acetylcysteine on curcumin-induced vacuolization and post-treatment recovery in NF1-deficient Schwann cells. (**A**) Quantification of vacuolated NF1-deficient Schwann cells following treatment with vehicle (DMSO), curcumin 20 μM, NAC 2 mM plus curcumin 20 μM, or NAC 2 mM alone. Cells were pretreated with NAC for 18 h and subsequently exposed to curcumin for 24 h in the continued presence of NAC. Vacuolization was scored and expressed according to the criteria described in Figure 1B. At least 187 cells per condition were scored in each independent experiment. (**B**) Quantification and representative images of post-treatment recovery after compound removal, under the indicated conditions, using the washout protocol described in Figure 2C. Recovery was assessed by crystal violet staining, and retained crystal violet was quantified by measuring absorbance at 595 nm. Values were normalized to DMSO-treated controls. Data are shown as mean ± SD from three independent experiments. Statistical significance was calculated for the comparisons indicated in the figure. **** *p* < 0.0001, one way ANOVA corrected for multiple comparisons using Tukey method.

The NF1-associated differences in selected antioxidant-response proteins prompted us to assess whether the antioxidant compound N-acetylcysteine (NAC) could modulate the vacuolizing response of NF1-deficient Schwann cells to curcumin. Cells were pretreated with NAC and subsequently exposed to curcumin in the continued presence of NAC. Whereas curcumin alone induced extensive cytoplasmic vacuolization, NAC pretreatment reduced the proportion of vacuolated cells to near-basal levels (Figure 4A). NAC alone did not induce appreciable vacuolization. The effect of NAC on curcumin-induced vacuolization was confirmed in the additional patient-derived NF1-deficient Schwann-cell line (Supplementary Figure S3).

Next, we assessed whether NAC also improved long-term recovery following curcumin removal. Cells previously exposed to curcumin alone showed minimal post-treatment recovery, whereas cells exposed to curcumin in the presence of NAC showed substantial recovery (Figure 4B). Thus, NAC strongly reduced curcumin-induced vacuolization, and this reduction was accompanied by an improvement in long-term proliferative recovery.

### 2.5. Acute NF1 Silencing Enhances Curcumin-Induced Vacuolization in an Independent Cellular Background

We next asked whether NF1 status affects susceptibility to the vacuolizing response to curcumin. As an initial comparison, NF1-proficient Schwann cells exposed to the same treatment conditions showed only a markedly attenuated vacuolizing response which did not reach statistical significance compared with vehicle-treated cells (Supplementary Figure S4), supporting the association between NF1 deficiency and susceptibility to curcumin-induced vacuolization.

**Figure 5:**
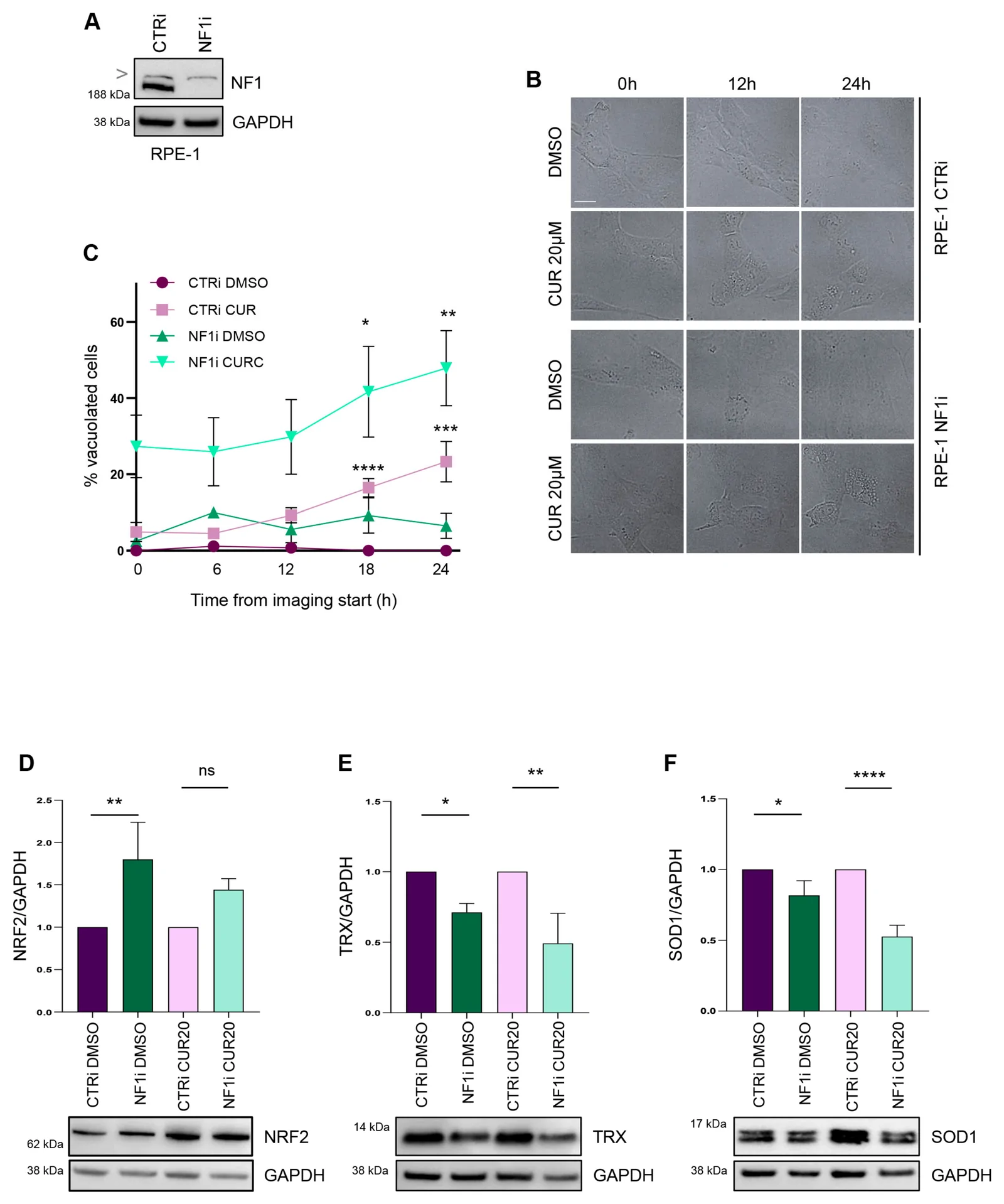
Acute NF1 silencing enhances curcumin-induced vacuolization in an independent cellular background. (**A**) Representative WB showing NF1 knockdown efficiency in hTERT RPE-1 cells transfected with control or NF1-targeting siRNAs, 48 h after transfection. GAPDH was used as a loading control. The asterisk indicates a non-specific band. (**B**) Representative images of hTERT RPE-1 cells transfected with control siRNAs (CTRi) or NF1-targeting siRNAs (NF1i) and treated with vehicle or curcumin 20 μM for 24 h. Images correspond to representative frames from time-lapse imaging experiments. Scale bars, 20 μm. (**C**) Time-lapse quantification of vacuolated hTERT RPE-1 cells following transfection with control or NF1-targeting siRNAs. Cells were treated with vehicle or curcumin 20 μM 24 h after transfection, and imaging was initiated 1 h after treatment. The percentage of vacuolated cells was calculated as in Figure 1C and data shown as mean ± SEM from three independent experiments. At least 108 cells were analyzed per condition across the three experiments. Statistical significance was calculated relative to vehicle-treated control at the indicated time points. * *p* = 0.0248; ** *p* = 0.0034; *** *p* = 0.0001; **** *p* < 0.0001, two way ANOVA corrected for multiple comparisons using Sidak method. (**D**–**F**) Representative WBs and corresponding densitometric quantification of selected proteins in the indicated cells treated with vehicle or curcumin 20 μM for 24 h. Protein levels were normalized to GAPDH and expressed relative to control-siRNA cells. Data are shown as mean ± SD from three independent experiments. ns, not significant; * *p* < 0.05; ** *p* < 0.01; **** *p* < 0.0001.

To further test the contribution of NF1 in an independent cellular background, we acutely silenced NF1 in hTERT RPE-1 cells and monitored their response to curcumin. siRNA-mediated NF1 knockdown enhanced curcumin-induced vacuolization compared with control-transfected cells, as shown by representative imaging and time-lapse quantification of vacuolated cells over time (Figure 5A–C).

We then examined whether this phenotype was accompanied by differences in the antioxidant effectors. In hTERT RPE-1 cells, acute NF1 silencing was associated with lower basal levels of TRX and SOD1 compared with control-transfected cells. Following curcumin exposure, TRX and SOD1 levels remained lower in NF1-silenced than in control-transfected cells (Figure 5D,F). Thus, the differences in TRX and SOD1 associated with acute NF1 silencing were consistent in direction with those observed in NF1-deficient Schwann cells (Figure 5D,F).

Together with the markedly attenuated response of NF1-proficient Schwann cells, these findings show that acute reduction in NF1 expression enhances curcumin-induced vacuolization in an independent cellular background, further supporting a key contribution of NF1 status in susceptibility to vacuolizing stress.

## 3. Discussion

The present study identifies curcumin-induced vacuolization as a prominent and functionally relevant response to NF1-deficient Schwann cells. Although only modestly reflected in short-term viability assays, this phenotype was associated with marked impairment of long-term clonogenic recovery. Notably, NAC reduced vacuolization to near-basal levels, and this pronounced attenuation was accompanied by substantial recovery of post-treatment proliferative fitness. Moreover, acute NF1 silencing recapitulated the enhanced susceptibility to curcumin-induced vacuolization in an independent cellular background, further supporting a role for NF1 loss in this response.

This observation is relevant in the context of NF1 disease biology because the study was performed in patient-derived NF1-deficient Schwann cells, a disease-relevant cellular compartment for NF1-associated neurofibromas. Although neurofibromas are complex multicellular lesions, biallelic NF1 loss in Schwann-lineage cells is a key initiating event in lesion formation. Defining how NF1-deficient Schwann cells respond to curcumin therefore provides a framework for understanding the consequences of curcumin exposure in an NF1-relevant context.

The phenotype observed in NF1-deficient Schwann cells is most appropriately interpreted within a methuosis-like framework. Curcumin-induced vacuoles closely resembled those induced by a reference methuosis inducer and accumulated without prominent biochemical evidence of canonical caspase-dependent apoptosis under the conditions examined. This interpretation is consistent with the sustained RAS pathway activation caused by neurofibromin loss and with biological frameworks linking aberrant RAS signaling, macropinocytic processes, and methuosis-like responses. Nevertheless, vacuolizing phenotypes may encompass a spectrum of partially overlapping non-apoptotic stress responses and may not conform to discrete mechanistic categories [24,25,26,27]. Because the present study did not include ultrastructural or compartment-specific analyses, we use the term methuosis-like descriptively, without assigning the vacuoles to a specific organellar compartment.

A second major finding of this study is the association between NF1 status and susceptibility to curcumin-induced vacuolizing stress. NF1-proficient Schwann cells displayed a markedly attenuated vacuolizing response compared with their NF1-deficient counterparts, whereas acute NF1 silencing in hTERT RPE-1 cells enhanced curcumin-induced vacuolization in an independent cellular background. Together, these complementary observations support a key contribution of NF1 status in determining susceptibility to this phenotype. Although the downstream mechanisms remain to be defined, our findings extend the consequences associated with NF1 loss beyond proliferative signaling to include altered responses to vacuolizing stress. The NF1-proficient and NF1-deficient Schwann-cell models are not isogenic, and differences in their genetic or cellular backgrounds cannot be excluded as contributors to their distinct responses. Nevertheless, the concordant effect of acute NF1 depletion in an independent cellular background strengthens the association between reduced NF1 expression and enhanced vacuolization, although the absence of an siRNA-resistant NF1 rescue limits the causal interpretation of this experiment.

An important aspect of our findings is the dissociation between short-term and long-term readouts. At early time points, curcumin induced extensive vacuolization while producing only a very limited reduction in the MTS-based short-term viability readout and no prominent PARP processing or accumulation of cleaved caspase-3 under the conditions analyzed. By comparison, prior curcumin exposure markedly impaired clonogenic recovery in two complementary long-term assay settings. This distinction is important because acute viability assays and classical apoptosis markers may not capture the full biological impact of vacuolizing non-canonical responses. In our system, the strongest functional consequence emerged in long-term recovery assays, revealing an association between the prominent vacuolizing response and durable loss of proliferative fitness in NF1-deficient Schwann cells.

Our data further show that susceptibility to curcumin-induced vacuolization occurs in the context of NF1-associated differences in selected antioxidant-response proteins. NF1-deficient Schwann cells displayed lower basal levels of NRF2, TRX, and SOD1 than NF1-proficient Schwann cells, and these differences remained evident following curcumin exposure. Although curcumin has been reported to regulate KEAP1-NRF2 signaling, its effects depend on cellular context, dose, exposure time, and the endpoint analyzed. In the present study, total cellular protein levels were assessed at 24 h; earlier or transient changes in protein stabilization, subcellular localization, or transcriptional activity were not examined. Thus, under the conditions analyzed, differences in the abundance of these selected antioxidant-response proteins were primarily associated with NF1 status, with no clear curcumin-dependent change. Acute NF1 silencing in hTERT RPE-1 cells was similarly associated with lower TRX and SOD1 levels under basal and curcumin-treated conditions, consistent with the differences observed in Schwann cells.

The antioxidant-response context is consistent with independent evidence that NF1-null Schwann cells undergo broad metabolic and mitochondrial rewiring, including alterations in oxidative phosphorylation, mitochondrial function, glycolytic features, and oxidative stress-related programs [8]. Notably, proteomic profiling of immortalized NF1 Schwann-cell models identified reduced expression of the mitochondrial antioxidant enzyme SOD2 in NF1-null cells, further supporting the existence of NF1-dependent differences in redox-associated cellular programs [8]. A broader relationship between NF1 loss and altered metabolic and redox homeostasis has also emerged from model organisms, including Drosophila, extending the biological consequences of NF1 loss beyond a strictly RAS-centric framework [28,29]. The NAC experiments complement these associative findings by showing that NAC markedly attenuated curcumin-induced vacuolization and improved clonogenic recovery after treatment withdrawal, supporting a possible contribution of redox-sensitive processes. However, because NAC can affect multiple redox-independent processes, further studies will be required to define the specific molecular pathways underlying curcumin-induced vacuolization and to determine the contribution of redox-sensitive processes to this phenotype in NF1-deficient Schwann cells. The attenuation of vacuolization by NAC was reproduced in a second patient-derived NF1-deficient Schwann-cell line, supporting the robustness of this response. Antioxidant-response protein levels and long-term clonogenic recovery, however, were examined primarily in the principal model.

From a translational perspective, curcumin is of interest in this context because of its long-standing human exposure and pleiotropic biological activities. In the present study, curcumin revealed an NF1-associated susceptibility to vacuolizing stress and impaired clonogenic recovery in disease-relevant cells. Previous nutritional and clinical investigations provided part of the rationale for examining curcumin in NF1 ([12]; NCT05363267), but the concentrations and exposure conditions used in this in vitro study cannot be directly extrapolated to patients. Further evaluation of achievable exposure, pharmacokinetics, selectivity, tolerability, formulation, and activity in additional disease-relevant and in vivo models would be required to assess the therapeutic relevance of this response.

Together, these findings identify heightened susceptibility to curcumin-induced vacuolizing stress, accompanied by impaired clonogenic recovery, as a previously underappreciated feature of the NF1-deficient cellular state and support the broader investigation of vacuolizing stress as an NF1-associated cellular vulnerability.

## 4. Materials and Methods

### 4.1. Cell Lines and Culture Conditions

Human immortalized Schwann-cell models were used to compare NF1-proficient and NF1-deficient backgrounds. NF1-proficient Schwann cells (hTERT NF1+/+ ipn02.3 2λ; ATCC CRL-3392) and NF1-deficient patient-derived Schwann cells (hTERT NF1−/− ipNF95.6; ATCC CRL-3389) were used as principal models. An additional patient-derived NF1-deficient Schwann-cell line (hTERT NF1−/− ipNF05.5; ATCC CRL-3388) was used for supportive validation experiments. Schwann cells were cultured in high-glucose DMEM GlutaMAX medium (#10566016, Life Technologies, Waltham, MA, USA) supplemented with 10% heat-inactivated fetal bovine serum (FBS, #A5256701, Life Technologies) and 1% penicillin-streptomycin (#15140122, Life Technologies). hTERT RPE-1 retinal pigment epithelial cells, kindly provided by Dr. Guarguaglini, were used for siRNA-mediated NF1 silencing experiments. hTERT RPE-1 cells were cultured in DMEM/F-12 (#12634010, Life Technologies) supplemented with 10% heat-inactivated FBS and 1% penicillin-streptomycin and 1% GlutaMAX (#3505061, Life Technologies). All cells were maintained under standard humidified conditions at 37 °C and 5% CO_2_ and were routinely tested for mycoplasma contamination.

### 4.2. Drug Treatments

Curcumin (#218580100 Life Technologies) was dissolved in DMSO (#D5879, Sigma-Aldrich/Merck, Darmstadt, Germany), aliquoted, stored protected from light, and used from single-use aliquots at the indicated concentrations. Vehicle-treated cells received equivalent volumes of DMSO. For phenotypic comparison, a reference methuosis inducer (methuosis Inducer 1/HZX-02-059, #HY-112440, Medchem Express, Monmouth Junction, NJ, USA) was used at the indicated concentration as a comparator for vacuolizing morphology.

NAC (#A9165, Sigma-Aldrich/Merck) was dissolved in sterile water. Cells were pretreated with NAC at 2 mM for 18h and subsequently exposed to curcumin in the continued presence of NAC for 24h. Parallel cultures received vehicle, curcumin alone, or NAC alone.

For apoptosis-control experiments, an apoptosis-inducing positive control was included in parallel (5 µM doxorubicin, #D1515 Sigma-Aldrich).

### 4.3. Morphological Assessment

For morphological analyses, cells were seeded in imaging-compatible culture plates and exposed to vehicle, curcumin, or reference compounds. Images were acquired at the indicated time points using a Nexcope NIB610-FL microscope (Nexcope, Ningbo, China) or an Eclipse Ti inverted microscope (Nikon, Tokyo, Japan). Multiple non-overlapping fields were randomly selected and acquired while blinded to the experimental condition. Cells displaying two or more prominent cytoplasmic vacuoles were manually scored as vacuolated, using a predefined criterion intended to exclude occasional isolated vacuole-like structures. The number of cells analyzed is reported in the corresponding figure legends.

### 4.4. Live-Cell Imaging

For time-lapse analyses, Schwann cells were cultured in DMEM high glucose without phenol red (#31053028, Life Technologies) and hTERT RPE-1 cells were cultured in DMEM/F-12 without phenol red (#21041025, Life Technologies) with 10% FBS and were seeded on 15μ-Slide 8 well (#80826, ibiTreat, Ibidi, Gräfelfing, Germany), positioned in a microscope stage incubator (Basic WJ, Okolab, Pozzuoli, Naples, Italy) at 37 °C and 5% CO2 and observed under an Eclipse Ti inverted microscope using a Plan Apo 40× objective (Nikon). DIC images were acquired every 20 min over 36h using a Clara camera (ANDOR Technology, Belfast, UK), and the NIS-Elements AR 3.22 software (Nikon). Movie processing and analysis were performed with NIS-Elements HC 5.02 (Nikon).

### 4.5. Viability Assay

Short-term viability was assessed 24 h after curcumin exposure using a metabolic viability assay (#G3580, CellTiter 96^®^ AQueous One Solution Cell Proliferation Assay, MTS; Promega, Madison, WI, USA) according to the manufacturer’s instructions. Signal was measured spectrophotometrically and normalized to vehicle-treated controls.

### 4.6. Clonogenic Recovery Assays

Long-term consequences of curcumin exposure were assessed using two complementary clonogenic approaches. In the first setting, cells were treated with curcumin, counted, and replated at defined densities (10,000 cells/well in 6-well plates); colony outgrowth was then assessed 8 days after replating. In the second setting, cells were exposed to curcumin, washed, and returned to fresh drug-free medium, after which recovery and colony formation were assessed 6 days after washing. Colonies were fixed and stained with 0.5% crystal violet in 25% ethanol/PBS for 20 min at room temperature, and imaged. Crystal violet retained by the colonies was subsequently solubilized in 10% acetic acid for 20 min at room temperature, and colony staining was quantified by measuring absorbance at 595 nm using a CLARIOstar microplate reader (BMG LABTECH GmbH, Ortenberg, Germany).

### 4.7. Protein Extraction and WB

Whole-cell lysates were prepared in lysis buffer (50 mM Tris-HCl pH 8, 150 mM NaCl, 0.5% sodium deoxycholate, 0.1% SDS, 1% NP40, 1 mM EDTA) supplemented with protease and phosphatase inhibitors (#11697498001, Roche, Basel, Switzerland). Proteins were resolved using Bolt Novex Bis-Tris Gels 4–12% gradient (Life Technologies). Membranes were probed with the following antibodies: anti-NF1 #sc-376886 (1:200) by Santa Cruz Biotechnology (Dallas, TX, USA), anti-PARP #10872500 (1:1000) by Roche, anti-NRF2 #PA5-8804 (1:1000) by Life Technologies, anti-TRX #EPR6110 (1:1000) by Abcam (Cambridge, UK), anti-SOD1 #TA50495 (1:1000) by OriGene Technologies, (Rockville, MD, USA), anti-cleaved caspase-3 (Asp175) #9661 (1:1000) by Cell Signaling Technology (Danvers, MA, USA) and anti-GAPDH-HRP #MA5-15738-HRP (1:5000) by Life Technologies. Bound antibodies were detected by chemiluminescence using WesternBright ECL (#K12045D50 Advansta, San Jose, CA, USA), and band intensities were quantified by densitometry with Image Lab software 5.0 (Bio-Rad, Hercules, CA, USA). Full length uncropped original WB were reported in Supplementary File S1.

### 4.8. siRNA-Mediated NF1 Silencing

For acute NF1 reduction, hTERT RPE-1 cells were transfected with a pool of validated NF1-targeting Stealth siRNAs (20 nM total final concentration; a mixture of three siRNAs with the following sequences: NF1HSS181496, 5′-AAACAAGGCAGUCAAUCAUUAGAUC-3′; NF1HSS181497, 5′-CAAGAAGCUAUAAGUAUCUUCUCUU-3′; and NF1HSS181498, 5′-ACUCCUGAAGGAAACAGCAUUUAAA-3′; Life Technologies) or universal negative-control siRNAs (#462001, Life Technologies) using lipofectamine RNAiMAX reagent (#13778075, Life Technologies), according to the manufacturer’s protocol. Knockdown efficiency was verified by WB.

### 4.9. Statistical Analysis

Data are presented as mean ± Standard Deviation (SD) for endpoint analyses and as mean ± Standard Error of the Mean (SEM) for time-course analyses, as specified in the corresponding figure legends. Unless otherwise indicated, data were obtained from three independent biological replicates. For image-based quantification, multiple fields per condition were analyzed, and the number of cells scored per experiment is indicated in the corresponding figure legends. For time-lapse analyses, multiple cells in several fields were followed longitudinally within each experiment. The percentage of vacuolated cells was calculated separately at each time point for each independent biological experiment. Data were then summarized across experiments, with the biological experiment considered the unit of replication. Statistical analyses were performed using GraphPad Prism version 8.0.2 (GraphPad Software, San Diego, CA, USA). Comparisons between two groups were performed using two-tailed unpaired Student’s *t*-tests. Experiments involving more than two conditions were analyzed using one-way ANOVA followed by the multiple-comparison procedure specified in the corresponding figure legend, according to the experimental design and comparisons of interest. *p* < 0.05 was considered statistically significant.

## Data Availability

The original contributions presented in this study are included in the article/Supplementary Materials. Further inquiries can be directed to the corresponding author.

## Supplementary Materials

The following supporting information can be downloaded at: https://www.mdpi.com/article/10.3390/cells15201904/s1, Supplementary Figure S1. Curcumin-induced vacuolization in an independent NF1-deficient Schwann-cell model. (**A**) Representative WB of NF1 in NF1-proficient Schwann cells (hTERT NF1+/+ ipn02.3), the NF1-deficient Schwann-cell line used throughout the study (hTERT NF1−/− ipNF95.6), and an additional NF1-deficient Schwann-cell line used for validation experiments (hTERT NF1−/− ipNF05.5). GAPDH was used as a loading control. The arrow indicates a non-specific band. (**B**,**C**) Representative images and quantification of vacuolated cells in the additional patient-derived NF1-deficient Schwann-cell line (hTERT NF1−/− ipNF05.5) treated with vehicle or curcumin 20 μM for 24 h. Scale bars, 50 μm. Data are shown as mean ± SD from three independent experiments. Statistical significance was calculated relative to the vehicle-treated control. *** *p* = 0.0006, unpaired *t*-test. Supplementary Figure S2. Short-term MTS-based viability and apoptosis-associated markers in NF1-deficient Schwann cells following curcumin exposure. (**A**) NF1-deficient Schwann cells were treated with vehicle or indicated concentrations of curcumin for 24 h, and short-term viability was assessed using an MTS-based metabolic assay. Values were normalized to DMSO-treated controls. Data are shown as mean ± SD. Statistical significance was calculated relative to DMSO-treated controls. ns, not significant; ANOVA test corrected for multiple comparisons using Dunnett method. (**B**) Representative WB of PARP and cleaved caspase-3 in NF1-deficient Schwann cells (hTERT NF1−/− ipNF05.5) treated with DMSO or curcumin 20 μM for 24 h. A lysate from doxorubicin-treated HCT116 cancer cells was included on the same blot as a positive control for apoptosis to verify PARP and caspase-3 cleavage. GAPDH was used as loading control. Supplementary Figure S3. NAC attenuates curcumin-induced vacuolization in an additional NF1-deficient Schwann-cell model. Quantification of vacuolated cells in the additional patient–derived NF1-deficient Schwann cell line (hTERT NF1−/− ipNF05.5) following treatment with vehicle (DMSO), curcumin 20 mM, NAC 2 mM plus curcumin 20 mM, or NAC 2 mM alone. Cells were pretreated with NAC for 18 h and subsequently exposed to curcumin for 24 h in the continued presence of NAC, according to the protocol described for Figure 4A. Vacuolization was scored and expressed as in Figure 4A. At least 182 cells per condition were scored in each independent experiment. Data are shown as mean ± SD from three independent experiments. Statistical significance was calculated for the comparisons indicated in the figure. **** *p* < 0.0001, one way ANOVA corrected for multiple comparisons using Tukey method. Supplementary Figure S4. NF1-proficient Schwann cells show attenuated vacuolization following curcumin exposure. (**A**) Representative images of NF1-proficient Schwann cells treated with vehicle, curcumin 20 μM, or the reference methuosis inducer 1 500 nM under the same experimental conditions used for NF1-deficient Schwann cells (Figure 1). Scale bars, 50 μm. (**B**) Time-lapse quantification of vacuole accumulation in NF1-proficient Schwann cells exposed to curcumin 20 μM or vehicle. Imaging was initiated 1 h after treatment. The percentage of vacuolated cells was calculated as in Figure 1C and data shown as mean ± SEM from three independent experiments. At least 76 cells were analyzed per condition across the three experiments. None of the comparisons reach statistical significance (*p* > 0.05) versus the vehicle-treated control group, as determined by a multiple-comparison *t*-test, corrected for multiple comparisons using Holm–Sidak method. Supplementary File S1. Uncropped WBs. Full, uncropped scans of the WBs shown in the main figures. The bands used in the corresponding figures are indicated.
