# Supplementary material for: Curcumin-Induced Vacuolizing Stress Reveals a Vulnerability Associated with NF1 Loss in Schwann Cells
Source: Cells. 2026 Oct 20;15(20):1904. doi: 10.3390/cells15201904 (PMC13703259; doi:10.3390/cells15201904)
Supplement: Supplementary file 1 [file cells-15-01904-s001.zip › cells-4580755-supplementary Figures.pdf]

FIGURE S1

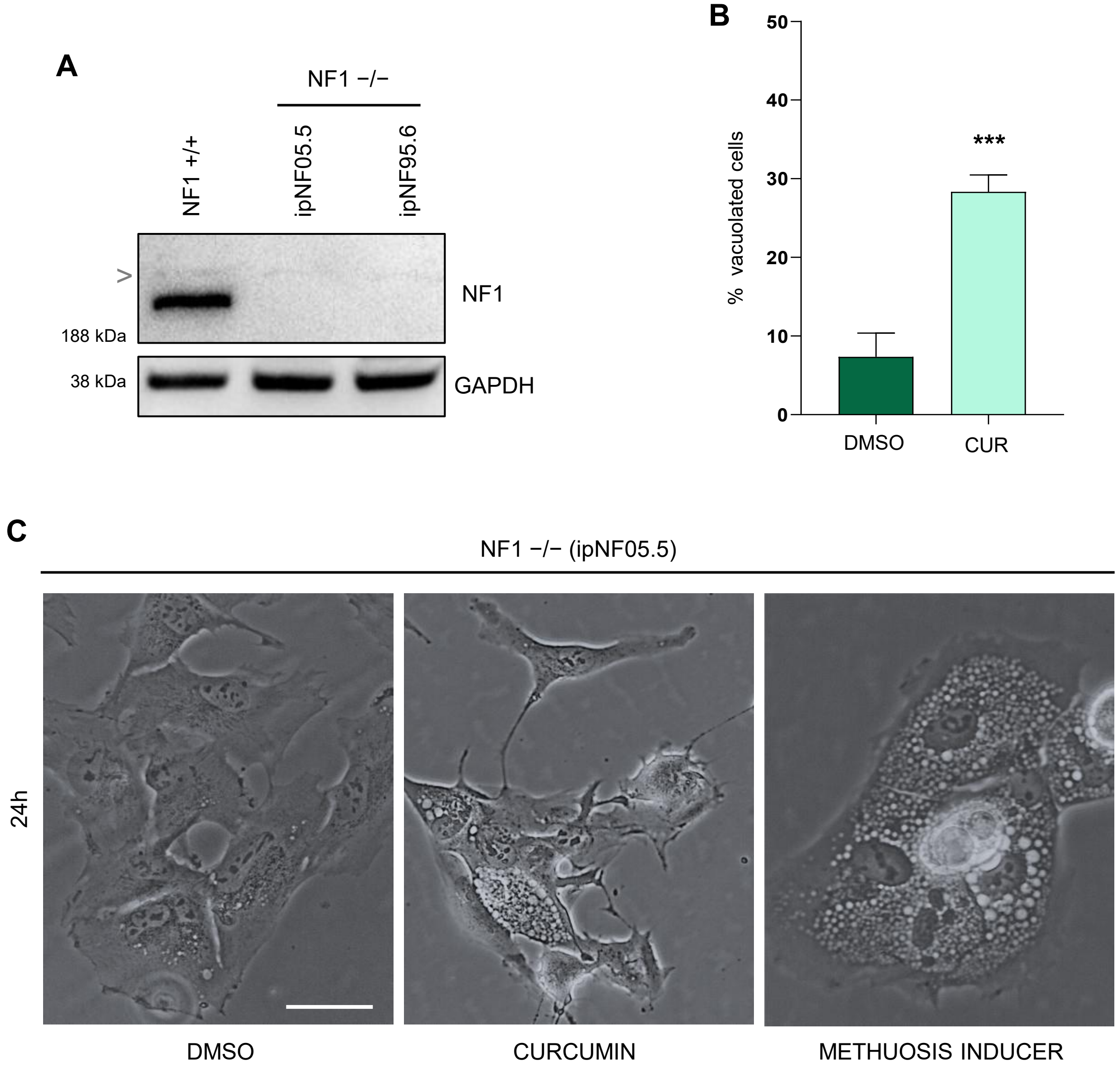
A
NF1 −/−
NF1 +/+
ipNF05.5
ipNF95.6
188 kDa
NF1
38 kDa
GAPDH
B
% vacuolated cells
50
40
30
20
10
0
***
DMSO
CUR
C
NF1 −/− (ipNF05.5)
24h
DMSO
CURCUMIN
METHUOSIS INDUCER

FIGURE S2

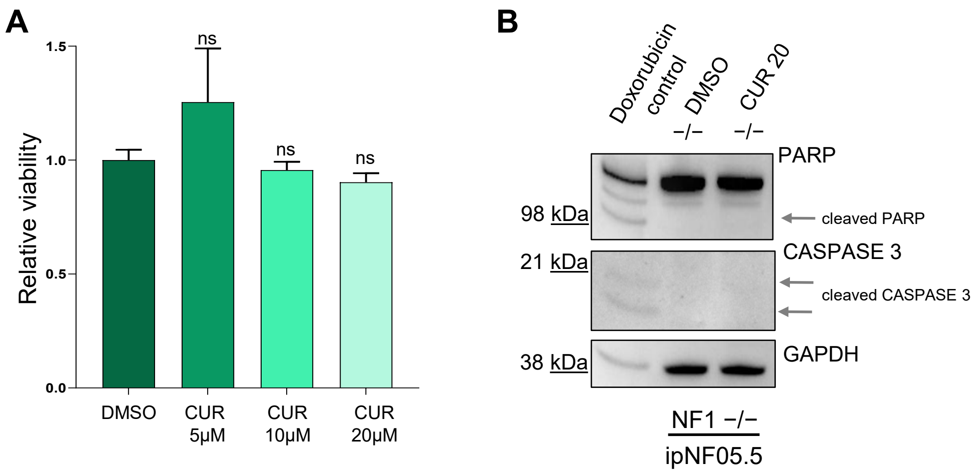
A
Relative viability
1.5
1.0
0.5
0.0
ns
ns
ns
DMSO
CUR 5μM
CUR 10μM
CUR 20μM
B
Doxorubicin control
DMSO −/−
CUR 20 −/−
PARP
98 kDa
cleaved PARP
21 kDa
CASPASE 3
cleaved CASPASE 3
38 kDa
GAPDH
NF1 −/−
ipNF05.5

FIGURE S3

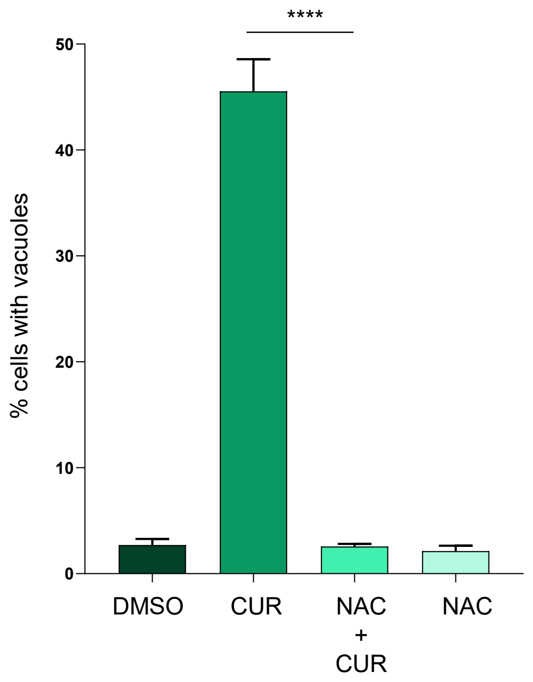

Figure S4

A

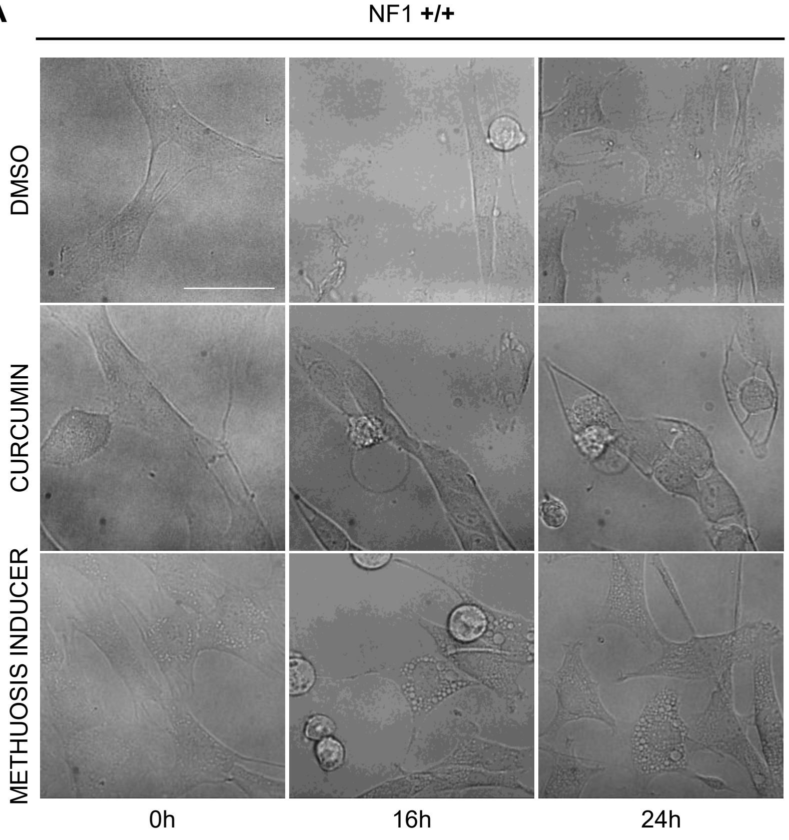
NF1 +/+
DMSO
CURCUMIN
METHUOSIS INDUCER
0h
16h
24h

B

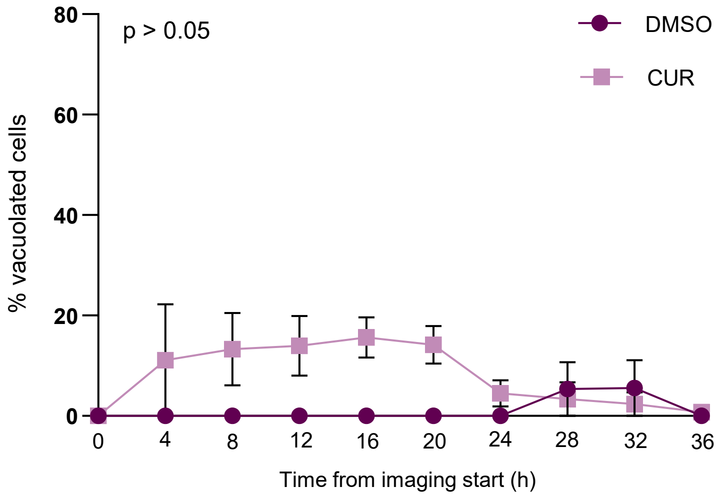
p > 0.05
DMSO
CUR
% vacuolated cells
80
60
40
20
0
0
4
8
12
16
20
24
28
32
36
Time from imaging start (h)
