# Supplementary material for: Curcumin-Induced Vacuolizing Stress Reveals a Vulnerability Associated with NF1 Loss in Schwann Cells
Source: Cells. 2026 Oct 20;15(20):1904. doi: 10.3390/cells15201904 (PMC13703259; doi:10.3390/cells15201904)

Uncropped WBs

Fig S1 A

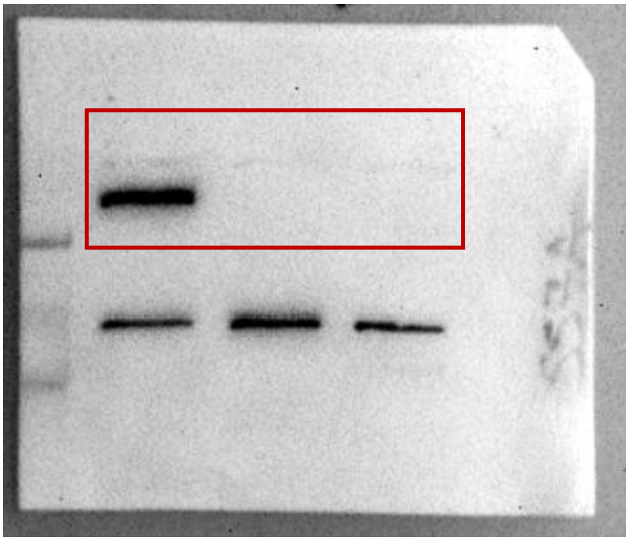

NF1

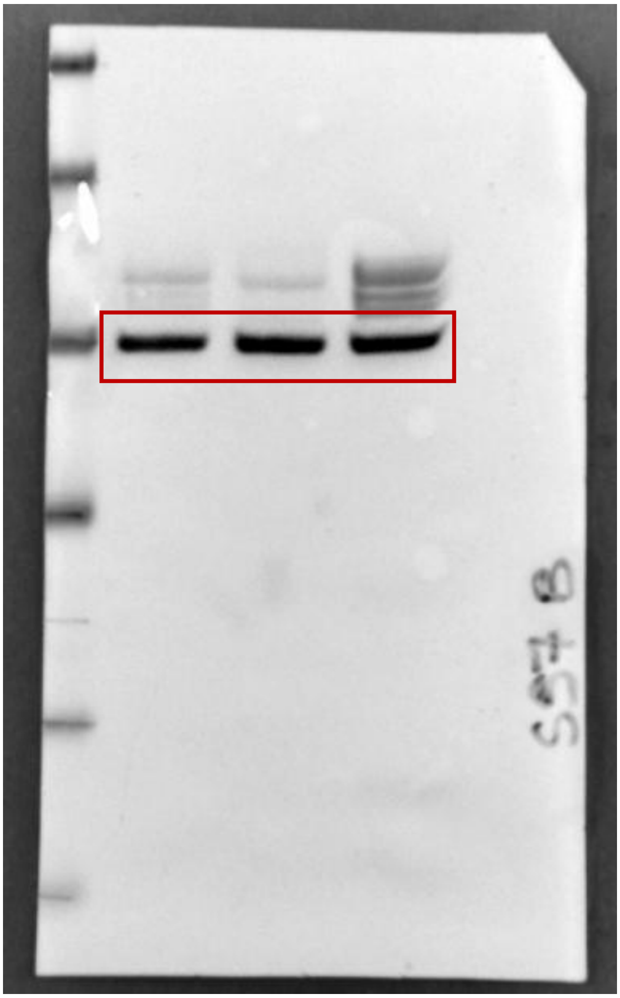

GAPDH

Fig 2 A

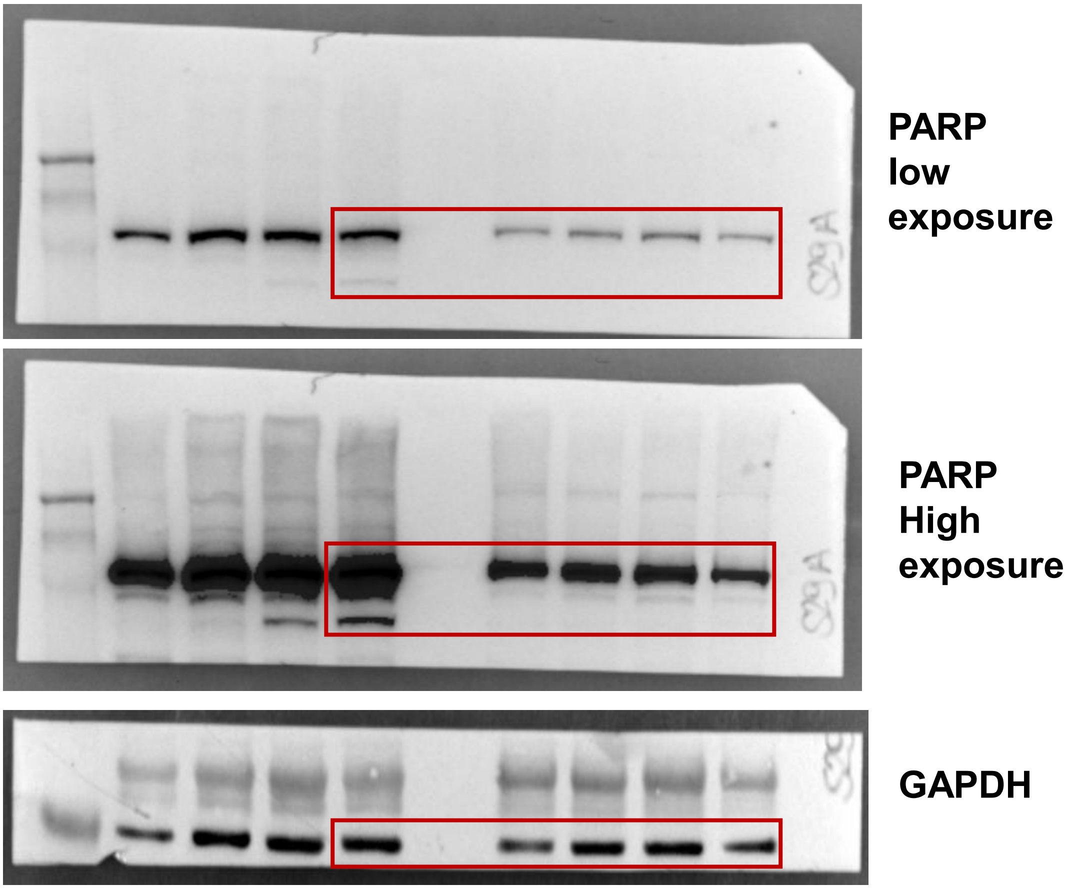

Fig S2 A

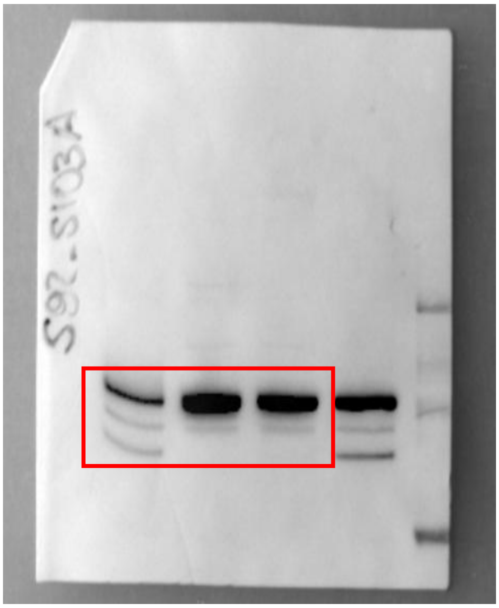

PARP

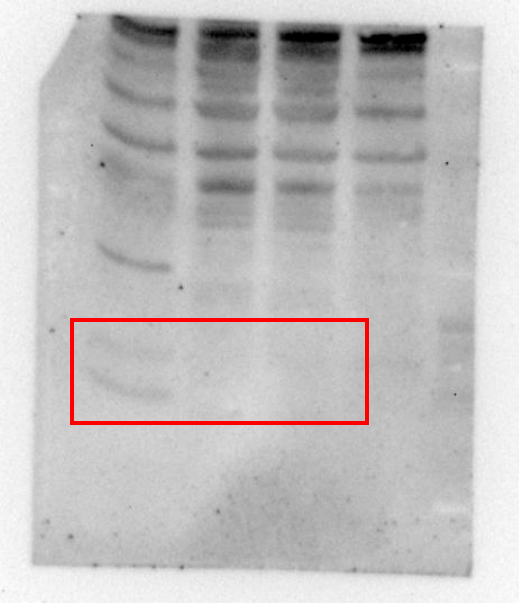

CASPASE 3 cleaved

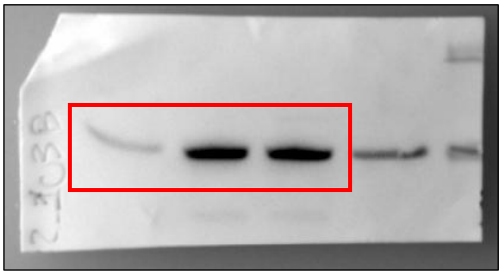

GAPDH

Fig 3 A

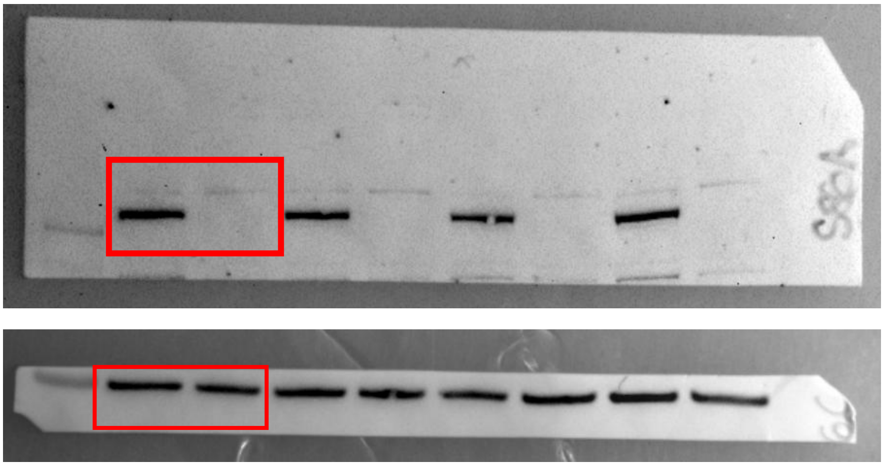

Fig 3 B

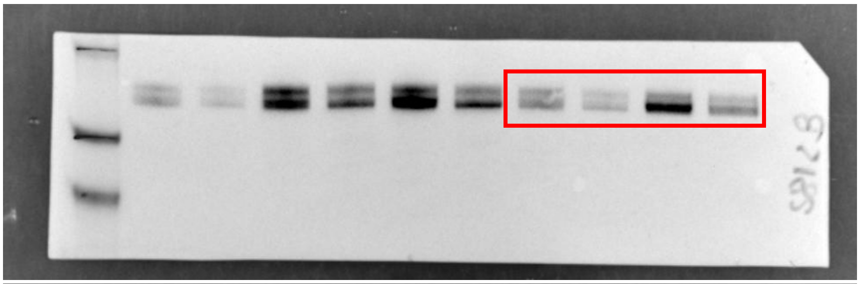

Fig 3 C

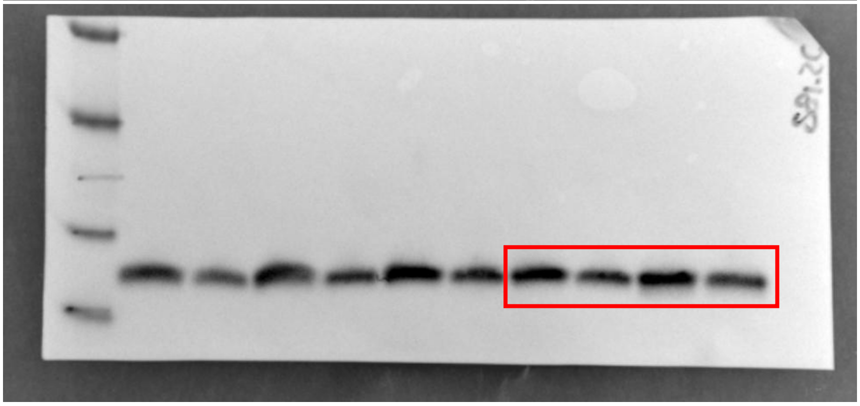

Fig 3 D

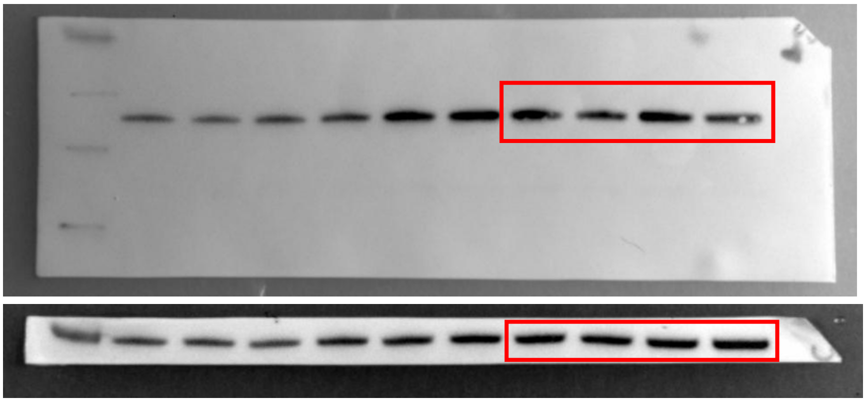

Fig 5 A

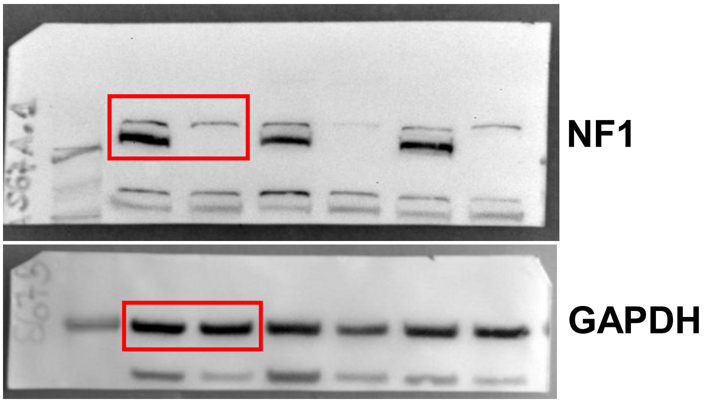

Fig 5D

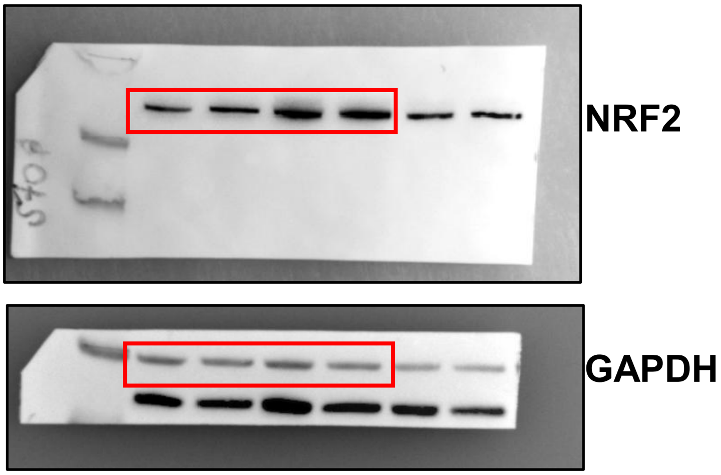

Fig 5E

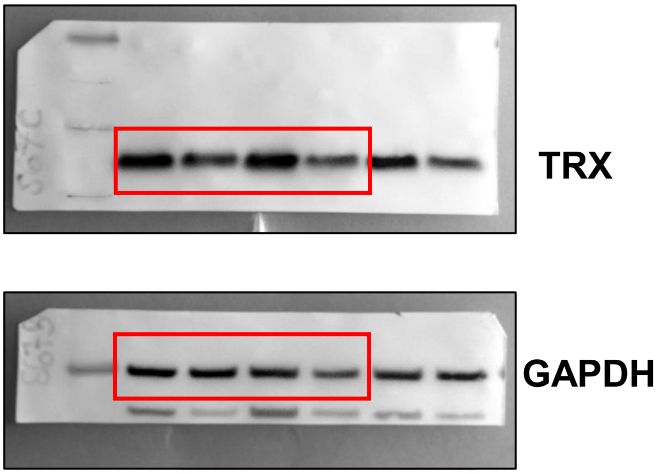

Fig 5 F

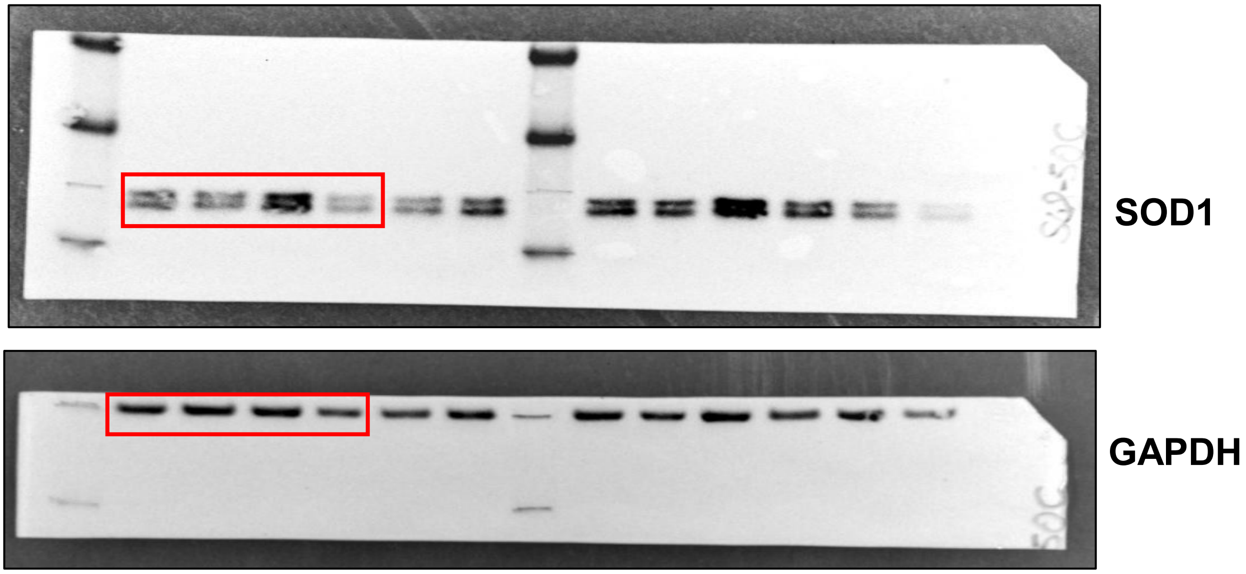

Supplement: Supplementary file 1 [file cells-15-01904-s001.zip › cells-4580755-supplementary File S1.pdf]
